# Patient medication management, understanding and adherence during the transition from hospital to outpatient care - a qualitative longitudinal study in polymorbid patients with type 2 diabetes

**DOI:** 10.1186/s12913-024-10784-9

**Published:** 2024-05-13

**Authors:** Léa Solh Dost, Giacomo Gastaldi, Marie P. Schneider

**Affiliations:** 1 School of Pharmaceutical Sciences, University of Geneva, Geneva, Switzerland; 2 Institute of Pharmaceutical Sciences of Western Switzerland, University of Geneva, Geneva, Switzerland; 3 Division of Endocrinology, Diabetes, Hypertension and Nutrition, Department of Medicine, Geneva University Hospitals, Geneva, Switzerland

**Keywords:** Continuity of care, Transition of care, Patient discharge, Medication management, Medication adherence, Qualitative research, Longitudinal studies, Patient-centered care, Interprofessional collaboration, Type 2 diabetes

## Abstract

**Background:**

Continuity of care is under great pressure during the transition from hospital to outpatient care. Medication changes during hospitalization may be poorly communicated and understood, compromising patient safety during the transition from hospital to home. The main aims of this study were to investigate the perspectives of patients with type 2 diabetes and multimorbidities on their medications from hospital discharge to outpatient care, and their healthcare journey through the outpatient healthcare system. In this article, we present the results focusing on patients’ perspectives of their medications from hospital to two months after discharge.

**Methods:**

Patients with type 2 diabetes, with at least two comorbidities and who returned home after discharge, were recruited during their hospitalization. A descriptive qualitative longitudinal research approach was adopted, with four in-depth semi-structured interviews per participant over a period of two months after discharge. Interviews were based on semi-structured guides, transcribed verbatim, and a thematic analysis was conducted.

**Results:**

Twenty-one participants were included from October 2020 to July 2021. Seventy-five interviews were conducted. Three main themes were identified: (A) Medication management, (B) Medication understanding, and (C) Medication adherence, during three periods: (1) Hospitalization, (2) Care transition, and (3) Outpatient care. Participants had varying levels of need for medication information and involvement in medication management during hospitalization and in outpatient care. The transition from hospital to autonomous medication management was difficult for most participants, who quickly returned to their routines with some participants experiencing difficulties in medication adherence.

**Conclusions:**

The transition from hospital to outpatient care is a challenging process during which discharged patients are vulnerable and are willing to take steps to better manage, understand, and adhere to their medications. The resulting tension between patients’ difficulties with their medications and lack of standardized healthcare support calls for interprofessional guidelines to better address patients’ needs, increase their safety, and standardize physicians’, pharmacists’, and nurses’ roles and responsibilities.

**Supplementary Information:**

The online version contains supplementary material available at 10.1186/s12913-024-10784-9.

## Introduction

Continuity of patient care is characterized as the collaborative engagement between the patient and their physician-led care team in the ongoing management of healthcare, with the mutual objective of delivering high-quality and cost-effective medical care [1]. Continuity of care is under great pressure during the transition of care from hospital to outpatient care, with a risk of compromising patients’ safety [2, 3]. The early post-discharge period is a high-risk and fragile transition: once discharged, one in five patients experience at least one adverse event during the first three weeks following discharge, and more than half of these adverse events are drug-related [4, 5]. A retrospective study examining all discharged patients showed that adverse drug events (ADEs) account for up to 20% of 30-day hospital emergency readmissions [6]. During hospitalization, patients’ medications are generally modified, with an average of nearly four medication changes per patient [7]. Information regarding medications such as medication changes, the expected effect, side effects, and instructions for use are frequently poorly communicated to patients during hospitalization and at discharge [8–11]. Between 20 and 60% of discharged patients lack knowledge of their medications [12, 13]. Consideration of patients’ needs and their active engagement in decision-making during hospitalization regarding their medications are often lacking [11, 14, 15]. This can lead to unsafe discharge and contribute to medication adherence difficulties, such as non-implementation of newly prescribed medications [16, 17].

Patients with multiple comorbidities and polypharmacy are at higher risk of ADE [18]. Type 2 diabetes is one of the chronic health conditions most frequently associated with comorbidities and patients with type 2 diabetes often lack care continuum [19–21]. The prevalence of patients hospitalized with type 2 diabetes can exceed 40% [22] and these patients are at higher risk for readmission due to their comorbidities and their medications, such as insulin and oral hypoglycemic agents [23–25].

Interventions and strategies to improve patient care and safety at transition have shown mixed results worldwide in reducing cost, rehospitalization, ADE, and non-adherence [26–35]. However, interventions that are patient-centered, with a patient follow-up and led by interprofessional healthcare teams showed promising results [34–36]. Most of these interventions have not been implemented routinely due to the extensive time to translate research into practice and the lack of hybrid implementation studies [37–41]. In addition, patient-reported outcomes and perspectives have rarely been considered, yet patients’ involvement is essential for seamless and integrated care [42, 43]. Interprofessional collaboration in which patients are full members of the interprofessional team, is still in its infancy in outpatient care [44]. Barriers and facilitators regarding medications at the transition of care have been explored in multiple qualitative studies at one given time in a given setting (e.g., at discharge, one-month post-discharge) [8, 45–48]. However, few studies have adopted a holistic methodology from the hospital to the outpatient setting to explore changes in patients’ perspectives over time [49–51]. Finally, little is known about whether, how, and when patients return to their daily routine following hospitalization and the impact of hospitalization weeks after discharge.

In Switzerland, continuity of care after hospital discharge is still poorly documented, both in terms of contextual analysis and interventional studies, and is mainly conducted in the hospital setting [31, 35, 52–56]. The first step of an implementation science approach is to perform a contextual analysis to set up effective interventions adapted to patients’ needs and aligned to healthcare professionals’ activities in a specific context [41, 57]. Therefore, the main aims of this study were to investigate the perspectives of patients with type 2 diabetes and multimorbidities on their medications from hospital discharge to outpatient care, and on their healthcare journey through the outpatient healthcare system. In this article, we present the results focusing on patients’ perspectives of their medications from hospital to two months after discharge.

## Methods

### Study design

**Fig. 1:**
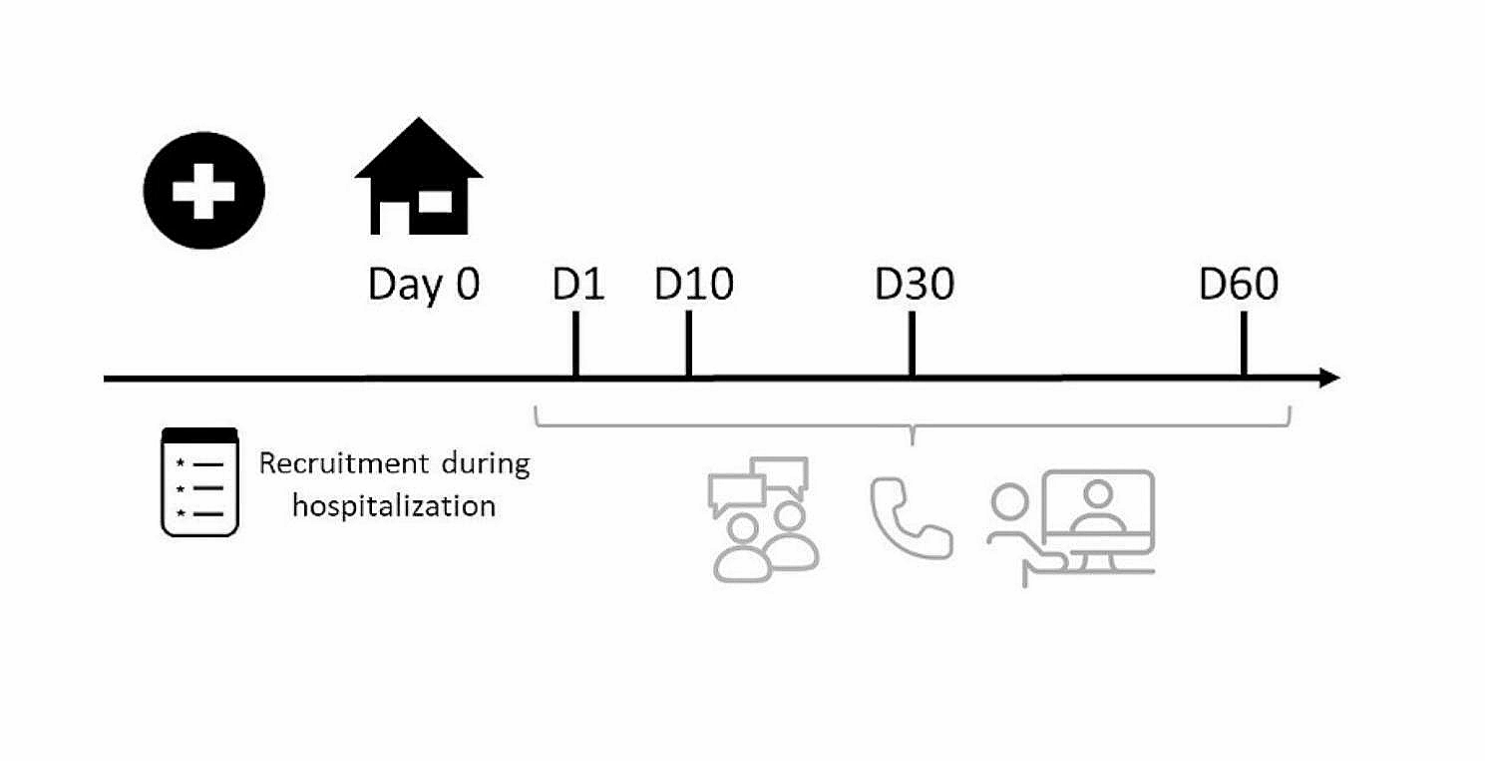
Study flowchart

This qualitative longitudinal study, conducted from October 2020 to July 2021, used a qualitative descriptive methodology through four consecutive in-depth semi-structured interviews per participant at three, 10-, 30- and 60-days post-discharge, as illustrated in Fig. 1. Longitudinal qualitative research is characterized by qualitative data collection at different points in time and focuses on temporality, such as time and change [58, 59]. Qualitative descriptive studies aim to explore and describe the depth and complexity of human experiences or phenomena [60–62]. We focused our qualitative study on the 60 first days after discharge as this period is considered highly vulnerable and because studies often use 30- or 60-days readmission as an outcome measure [5, 63].

This qualitative study follows the Consolidated Criteria for Reporting Qualitative Research (COREQ). Ethics committee approval was sought and granted by the Cantonal Research Ethics Commission, Geneva (CCER) (2020 − 01779).

### Settings

Recruitment took place during participants’ hospitalization in the general internal medicine divisions at the Geneva University Hospitals in the canton of Geneva (500 000 inhabitants), Switzerland. Interviews took place at participants’ homes, in a private office at the University of Geneva, by telephone or by secure video call, according to participants’ preference. Informal caregivers could also participate alongside the participants.

### Researcher characteristics

All the researchers were trained in qualitative studies. The diabetologist and researcher (GG) who enrolled the patients in the study was involved directly or indirectly (advice asked to the Geneva University Hospital diabetes team of which he was a part) for most participants’ care during hospitalization. LS (Ph.D. student and community pharmacist) was unknown to participants and presented herself during hospitalization as a “researcher” and not as a healthcare professional to avoid any risk of influencing participants’ answers. This study was not interventional, and the interviewer (LS) invited participants to contact a healthcare professional for any questions related to their medication or medical issues.

### Population and sampling strategy

Patients with type 2 diabetes were chosen as an example population to describe polypharmacy patients as these patients usually have several health issues and polypharmacy [20, 22, 25]. Inclusions criteria for the study were: adult patients with type 2 diabetes, with at least two other comorbidities, hospitalized for at least three days in a general internal medicine ward, with a minimum of one medication change during hospital stay, and who self-managed their medications once discharged home. Exclusion criteria were patients not reachable by telephone following discharge, unable to give consent (patients with schizophrenia, dementia, brain damage, or drug/alcohol misuse), and who could not communicate in French. A purposive sampling methodology was applied aiming to include participants with different ages, genders, types, and numbers of health conditions by listing participants’ characteristics in a double-entry table, available in Supplementary Material 1, until thematic saturation was reached. Thematic saturation was considered achieved when no new code or theme emerged and new data repeated previously coded information [64]. The participants were identified if they were hospitalized in the ward dedicated to diabetes care or when the diabetes team was contacted for advice. The senior ward physician (GG) screened eligible patients and the interviewer (LS) obtained written consent before hospital discharge.

### Data collection and instruments

Sociodemographic (age, gender, educational level, living arrangement) and clinical characteristics (reason for hospitalization, date of admission, health conditions, diabetes diagnosis, medications before and during hospitalization) were collected by interviewing participants before their discharge and by extracting participants’ data from electronic hospital files by GG and LS. Participants’ pharmacies were contacted with the participant’s consent to obtain medication records from the last three months if information regarding medications before hospitalization was missing in the hospital files.

Semi-structured interview guides for each interview (at three, 10-, 30- and 60-days post-discharge) were developed based on different theories and components of health behavior and medication adherence: the World Health Organization’s (WHO) five dimensions for adherence, the Information-Motivation-Behavioral skills model and the Social Cognitive Theory [65–67]. Each interview explored participants’ itinerary in the healthcare system and their perspectives on their medications. Regarding medications, the following themes were mentioned at each interview: changes in medications, patients’ understanding and implication; information on their medications, self-management of their medications, and patients’ medication adherence. Other aspects were mentioned in specific interviews: patients’ hospitalization and experience on their return home (interview 1), motivation (interviews 2 and 4), and patient’s feedback on the past two months (interview 4). Interview guides translated from French are available in Supplementary Material 2. The participants completed self-reported and self-administrated questionnaires at different interviews to obtain descriptive information on different factors that may affect medication management and adherence: self-report questionnaires on quality of life (EQ-5D-5 L) [68], literacy (Schooling-Opinion-Support questionnaire) [69], medication adherence (Adherence Visual Analogue Scale, A-VAS) [70] and Belief in Medication Questionnaire (BMQ) [71] were administered to each participant at the end of selected interviews to address the different factors that may affect medication management and adherence as well as to determine a trend of determinants over time. The BMQ contains two subscores: Specific-Necessity and Specific-Concerns, addressing respectively their perceived needs for their medications, and their concerns about adverse consequences associated with taking their medication [72].

### Data management

Informed consent forms, including consent to obtain health data, were securely stored in a private office at the University of Geneva. The participants’ identification key was protected by a password known only by MS and LS. Confidentiality was guaranteed by pseudonymization of participants’ information and audio-recordings were destroyed once analyzed. Sociodemographic and clinical characteristics, medication changes, and answers to questionnaires were securely collected by electronic case report forms (eCRFs) on RedCap®. Interviews were double audio-recorded and field notes were taken during interviews. Recorded interviews were manually transcribed verbatim in MAXQDA® (2018.2) by research assistants and LS and transcripts were validated for accuracy by LS. A random sample of 20% of questionnaires was checked for accuracy for the transcription from the paper questionnaires to the eCRFs. Recorded sequences with no link to the discussed topics were not transcribed and this was noted in the transcripts.

### Data analysis

A descriptive statistical analysis of sociodemographic, clinical characteristics and self-reported questionnaire data was carried out. A thematic analysis of transcripts was performed, as described by Braun and Clarke [73], by following six steps: raw data was read, text segments related to the study objectives were identified, text segments to create new categories were identified, similar or redundant categories were reduced and a model that integrated all significant categories was created. The analysis was conducted in parallel with patient enrolment to ensure data saturation. To ensure the validity of the coding method, transcripts were double coded independently and discussed by the research team until similar themes were obtained. The research group developed and validated an analysis grid, with which LS coded systematically the transcriptions and met regularly with the research team to discuss questions on data analysis and to ensure the quality of coding. The analysis was carried out in French, and the verbatims of interest cited in the manuscript were translated and validated by a native English-speaking researcher to preserve the meaning.

In this analysis, we used the term “healthcare professionals” when more than one profession could be involved in participants’ medication management. Otherwise, when a specific healthcare professional was involved, we used the designated profession (e.g. physicians, pharmacists).

### Patient and public involvement

During the development phase of the study, interview guides and questionnaires were reviewed for clarity and validity and adapted by two patient partners, with multiple health conditions and who experienced previously a hospital discharge. They are part of the HUG Patients Partners + 3P platform for research and patient and public involvement.

## Results

### Interviews and participants’ descriptions

A total of 75 interviews were conducted with 21 participants. In total, 31 patients were contacted, seven refused to participate (four at the project presentation and three at consent), two did not enter the selection criteria at discharge and one was unreachable after discharge. Among the 21 participants, 15 participated in all interviews, four in three interviews, one in two interviews, and one in one interview, due to scheduling constraints. Details regarding interviews and participants characteristics are presented in Tables 1 and 2.

**Table 1:** Interview characteristics

Interviews characteristics	N, %
Total number of interviews - Interview 1 - Interview 2 - Interview 3 - Interview 4	**75** 21 (28%) 19 (25,5%) 16 (21%) 19 (25,5%)
Duration (minutes), median (IQR)	41 (34-49)
Interview location - Home - Study center (university) - Telephone - Secure video call	26 (35%) 23 (30%) 15 (20%) 11 (15%)
Number of interviews with both participant and family caregiver	6 (8%)

**Table 2:** Participants characteristics

Demographics	N, %
Number of participants	21
Age (years), median (IQR)	63 (59-73)
Gender,	
- Men - Women	12 (57%) 9 (43%)
Reasons for hospitalization,	
- Type 2 diabetes - Myocardial infarction - Other cardiac reasons - Other reasons	9 (43%) 4 (19%) 5 (24%) 3 (14%)
Type 2 diabetes diagnosis,	
- Newly diagnosed (< 6 months or during hospitalization) - Diagnosed but not treated - > 6 months	9 (43%) 2 (10%) 10 (47%)
Educational level, *n* = *20/21*^*e*^	
- Obligatory schooling - Professional training - Professional college and university	7 (35%) 7 (35%) 6 (30%)
Help to read information provided by healthcare professionals,	
- never - rarely - sometimes	8 (38%) 6 (29%) 7 (33%)
Living arrangement,	
- Living with partner^a^ - Living with adult children^b^ - Living alone	11 (52%) 4 (19%) 6 (29%)
Self-rated health from 0-100 (EQ-VAS), median (IQR)	
- Interview 1 *n* = *20/21*^*e*^ - Interview 4 *n* = *15/19*^*e*^	60 (50-72.5) 75 (50-90)
Beliefs about medicines questionnaire^c^, *n* = *16/19*^*e*^	
- Necessity score^d^(5 items), median (IQR) - Concerns score^d^ (5 items), median (IQR)	4.4 (3-4) 2.9 (2-4)

^a^Married or in partnership

^b^There was no participant living with children under 18 years old

^c^The medication that each participant has the most questions about or has the most problems with

^d^5-point Likert scale varying from 1=“strongly disagree” to 5=”strongly agree”

^e^Missing data

The median length of time between hospital discharge and interviews 1,2,3 and 4 was 5 (IQR: 4–7), 14 (13-20), 35 (22-38), and 63 days (61-68), respectively. On average, by comparing medications at hospital admission and discharge, a median of 7 medication changes (IQR: 6–9, range:2;17) occurred per participant during hospitalization and a median of 7 changes (5–12) during the two months following discharge. Details regarding participants’ medications are described in Table 3.

**Table 3:** Description of participants’ medications

Medication characteristics	Median (IQR)
Medications *before hospitalization*	6 (3-7)
Medications *at discharge*	9 (7-12)
Medications *at 2 months post-discharge*	9 (6-10)
Medication changes during hospitalization^*a*^, Total medication changes during hospitalization, n	7 (6-9) 170
Types of changes during hospitalization (%)	
- Initiation^b^ - Interruption^*c*^ - Change in dosage^d^ - Change in regimen^e^ - Other changes (brand, formulation, etc.)	72% 15% 6% 3.5% 3.5%
Medication changes during follow-up Total medication changes during follow-up, n	7 (5-12) 201
Types of changes during follow-up (%)	
- Interruption - Initiation - Change in dosage - Change in regimen - Other changes (brand, formulation, etc.)	33% 26% 16% 9% 26%

^a^Comparison of medications before and after hospital admission

^b^Initiation of a new medication

^c^Stop of a medication

^d^Increase/decrease of drug dosage

^e^Increase/decrease of frequency of medication intake

Patient self-reported adherence over the past week for their three most challenging medications are available in Supplementary Material 3.

### Qualitative analysis

**Fig. 2:**
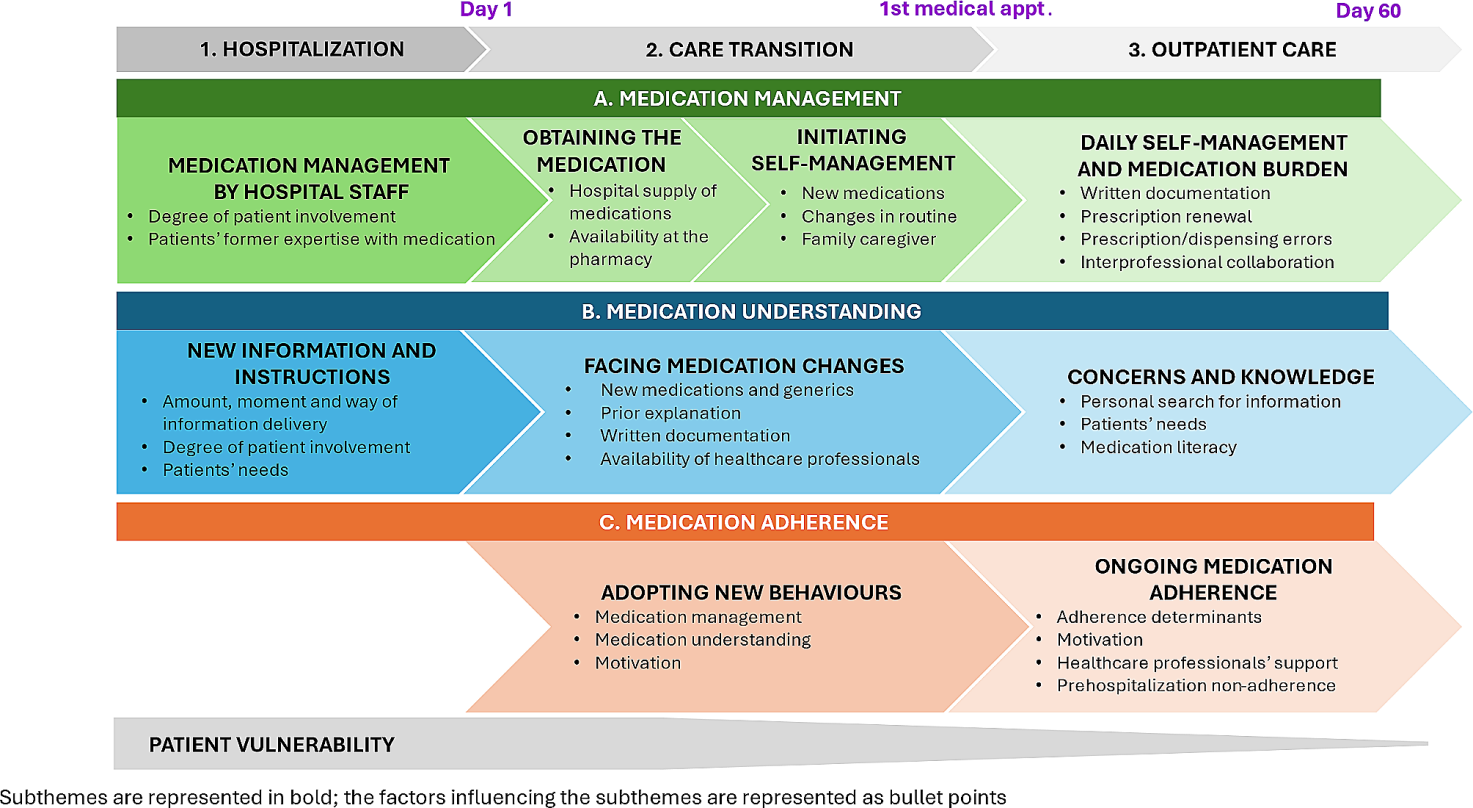
Participants’ medication management, understanding and adherence during hospitalization, care transition and outpatient care

We defined care transition as the period from discharge until the first medical appointment post-discharge, and outpatient care as the period starting after the first medical appointment. Data was organized into three key themes (A. Medication management, B. Medication understanding, and C. Medication adherence) divided into subthemes at three time points (1. Hospitalization, 2. Care transition and 3. Outpatient care). Figure 2 summarizes and illustrates the themes and subthemes with their influencing factors as bullet points.

### A. Medication management

#### A.1 Medication management during hospitalization: medication management by hospital staff

Medications during hospitalization were mainly managed by hospital healthcare professionals (i.e. nurses and physicians) with varying degrees of patient involvement: *“At the hospital, they prepared the medications for me. […] I didn’t even know what the packages looked like.” Participant 22; interview 1 (P22.1)* Some participants reported having therapeutic education sessions with specialized nurses and physicians, such as the explanation and demonstration of insulin injection and glucose monitoring. A patient reported that he was given the choice of several treatments and was involved in shared decision-making. Other participants had an active role in managing and optimizing dosages, such as rapid insulin, due to prior knowledge and use of medications before hospitalization.

#### A.2 Medication management at transition: obtaining the medication and initiating self-management

Once discharged, some participants had difficulties obtaining their medications at the pharmacy because some medications were not stored and had to be ordered, delaying medication initiation. To counter this problem upstream, a few participants were provided a 24-to-48-hour supply of medications at discharge. It was sometimes requested by the patient or suggested by the healthcare professionals but was not systematic. The transition from medication management by hospital staff to self-management was exhausting for most participants who were faced with a large amount of new information and changes in their medications: “*When I was in the hospital, I didn’t even realize all the changes. When I came back home, I took away the old medication packages and got out the new ones. And then I thought*: «*my God, all this…I didn’t know I had all these changes*»*” P2.1* Written documentation, such as the discharge prescription or dosage labels on medication packages, was helpful in managing their medication at home. Most participants used weekly pill organizers to manage their medications, which were either already used before hospitalization or were introduced post-discharge. The help of a family caregiver in managing and obtaining medications was reported as a facilitator.

#### A.3 Medication management in outpatient care: daily self-management and medication burden

A couple of days or weeks after discharge, most participants had acquired a routine so that medication management was less demanding, but the medication burden varied depending on the participants. For some, medication management became a simple action well implemented in their routine *(“It has become automatic”*, P23.4), while for others, the number of medications and the fact that the medications reminded them of the disease was a heavy burden to bear on a daily basis (“*During the first few days after getting out of the hospital, I thought I was going to do everything right. In the end, well [laughs] it’s complicated. I ended up not always taking the medication, not monitoring the blood sugar” P12.2)* To support medication self-management, some participants had written documentation such as treatment plans, medication lists, and pictures of their medication packages on their phones. Some participants had difficulties obtaining medications weeks after discharge as discharge prescriptions were not renewable and participants did not see their physician in time. Others had to visit multiple physicians to have their prescriptions updated. A few participants were faced with prescription or dispensing errors, such as prescribing or dispensing the wrong dosage, which affected medication management and decreased trust in healthcare professionals. In most cases, according to participants, the pharmacy staff worked in an interprofessional collaboration with physicians to provide new and updated prescriptions.

### B. Medication understanding

#### B.1 Medication understanding during hospitalization: new information and instructions

The amount of information received during hospitalization varied considerably among participants with some reporting having received too much, while others saying they received too little information regarding medication changes, the reason for changes, or for introducing new medications: *“They told me I had to take this medication all my life, but they didn’t tell me what the effects were or why I was taking it.” P5.3*

Hospitalization was seen by some participants as a vulnerable and tiring period during which they were less receptive to information. Information and explanations were generally given verbally, making it complicated for most participants to recall it. Some participants reported that hospital staff was attentive to their needs for information and used communication techniques such as teach-back (a way of checking understanding by asking participants to say in their own words what they need to know or do about their health or medications). Some participants were willing to be proactive in the understanding of their medications while others were more passive, had no specific needs for information, and did not see how they could be engaged more.

#### B.2 Medication understanding at transition: facing medication changes

At hospital discharge, the most challenging difficulty for participants was to understand the changes made regarding their medications. For newly diagnosed participants, the addition of new medications was more difficult to understand, whereas, for experienced participants, changes in known medications such as dosage modification, changes within a therapeutic class, and generic substitutions were the most difficult to understand. Not having been informed about changes caused confusion and misunderstanding. Therefore, medication reconciliation done by the patient was time-consuming, especially for participants with multiple medications: “*They didn’t tell me at all that they had changed my treatment completely. They just told me*: «*We’ve changed a few things. But it was the whole treatment*».*” P2.3* Written information, such as the discharge prescription, the discharge report (brief letter summarizing information about the hospitalization, given to the patient at discharge), or the label on the medication box (written by the pharmacist with instructions on dosage) helped them find or recall information about their medications and diagnoses. However, technical terms were used in hospital documentations and were not always understandable. For example, this participant said: “*On the prescription of valsartan, they wrote: ‘resume in the morning once profile…’[once hypertension profile allows]… I don’t know what that means.” P8.1* In addition, some documents were incomplete, as mentioned by a patient who did not have the insulin dosage mentioned on the hospital prescription. Some participants sought help from healthcare professionals, such as pharmacists, hospital physicians, or general practitioners a few days after discharge to review medications, answer questions, or obtain additional information.

#### B.3 Medication understanding in the outpatient care: concerns and knowledge

Weeks after discharge, most participants had concerns about the long-term use of their medications, their usefulness, and the possible risk of interactions or side effects. Some participants also reported having some lack of knowledge regarding indications, names, or how the medication worked: *“I don’t even know what Brilique® [ticagrelor, antiplatelet agent] is for. It’s for blood pressure, isn’t it?. I don’t know.” P11.4* According to participants, the main reasons for the lack of understanding were the lack of information at the time of prescribing and the large number of medications, making it difficult to search for information and remember it. Participants sought information from different healthcare professionals or by themselves, on package inserts, through the internet, or from family and friends. Others reported having had all the information needed or were not interested in having more information. In addition, participants with low medication literacy, such as non-native speakers or elderly people, struggled more with medication understanding and sought help from family caregivers or healthcare professionals, even weeks after discharge: “*I don’t understand French very well […] [The doctor] explained it very quickly…[…] I didn’t understand everything he was saying” P16.2*

### C. Medication adherence

#### C.2 Medication adherence at transition: adopting new behaviors

Medication adherence was not mentioned as a concern during hospitalization and a few participants reported difficulties in medication initiation once back home: *“I have an injection of Lantus® [insulin] in the morning, but obviously, the first day [after discharge], I forgot to do it because I was not used to it.” P23.1* Participants had to quickly adopt new behaviors in the first few days after discharge, especially for participants with few medications pre-hospitalization. The use of weekly pill organizers, alarms and specific storage space were reported as facilitators to support adherence. One patient did not initiate one of his medications because he did not understand the medication indication, and another patient took her old medications because she was used to them. Moreover, most participants experienced their hospitalization as a turning point, a time when they focused on their health, thought about the importance of their medications, and discussed any new lifestyle or dietary measures that might be implemented.

#### C.3 Medication adherence in outpatient care: ongoing medication adherence

More medication adherence difficulties appeared a few weeks after hospital discharge when most participants reported nonadherence behaviors, such as difficulties implementing the dosage regimen, or intentionally discontinuing the medication and modifying the medication regimen on their initiative. Determinants positively influencing medication adherence were the establishment of a routine; organizing medications in weekly pill-organizers; organizing pocket doses (medications for a short period that participants take with them when away from home); seeking support from family caregivers; using alarm clocks; and using specific storage places. Reasons for nonadherence were changes in daily routine; intake times that were not convenient for the patient; the large number of medications; and poor knowledge of the medication or side effects. Healthcare professionals’ assistance for medication management, such as the help of home nurses or pharmacists for the preparation of weekly pill-organizers, was requested by participants or offered by healthcare professionals to support medication adherence: “*I needed [a home nurse] to put my pills in the pillbox. […] I felt really weak […] and I was making mistakes. So, I’m very happy [the doctor] offered me [home care]. […] I have so many medications.” P22.3* Some participants who experienced prehospitalization non-adherence were more aware of their non-adherence and implemented strategies, such as modifying the timing of intake: *“I said to my doctor*: «*I forget one time out of two […], can I take them in the morning?* » *We looked it up and yes, I can take it in the morning.” P11.2* In contrast, some participants were still struggling with adherence difficulties that they had before hospitalization. Motivations for taking medications two months after discharge were to improve health, avoid complications, reduce symptoms, reduce the number of medications in the future or out of obligation: “*I force myself to take them because I want to get to the end of my diabetes, I want to reduce the number of pills as much as possible.” P14.2* After a few weeks post-hospitalization, for some participants, health and illness were no longer the priority because of other life imperatives (e.g., family or financial situation).

## Discussion

This longitudinal study provided a multi-faceted representation of how patients manage, understand, and adhere to their medications from hospital discharge to two months after discharge. Our findings highlighted the varying degree of participants’ involvement in managing their medications during their hospitalization, the individualized needs for information during and after hospitalization, the complicated transition from hospital to autonomous medication management, the adaptation of daily routines around medication once back home, and the adherence difficulties that surfaced in the outpatient care, with nonadherence prior to hospitalization being an indicator of the behavior after discharge. Finally, our results confirmed the lack of continuity in care and showed the lack of patient care standardization experienced by the participants during the transition from hospital to outpatient care.

This in-depth analysis of patients’ experiences reinforces common challenges identified in the existing literature such as the lack of personalized information [9–11], loss of autonomy during hospitalization [14, 74, 75], difficulties in obtaining medication at discharge [11, 45, 76] and challenges in understanding treatment modifications and generics substitution [11, 32, 77, 78]. Some of these studies were conducted during patients’ hospitalization [10, 75, 79] or up to 12 months after discharge [80, 81], but most studies focused on the few days following hospital discharge [9, 11, 14, 82]. Qualitative studies on medications at transition often focused on a specific topic, such as medication information, or a specific moment in time, and often included healthcare professionals, which muted patients’ voices [9–11, 47, 49]. Our qualitative longitudinal methodology was interested in capturing the temporal dynamics, in-depth narratives, and contextual nuances of patients’ medication experiences during transitions of care [59, 83]. This approach provided a comprehensive understanding of how patients’ perspectives and behaviors evolved over time, offering insights into the complex interactions of medication management, understanding and adherence, and turning points within their medication journeys. A qualitative longitudinal design was used by Fylan et al. to underline patients’ resilience in medication management during and after discharge, by Brandberg et al. to show the dynamic process of self-management during the 4 weeks post-discharge and by Lawton et al. to examine how patients with type 2 diabetes perceived their care after discharge over a period of four years [49–51]. Our study focused on the first two months following hospitalization and future studies should focus on following discharged and at-risk patients over a longer period, as “transitions of care do not comprise linear trajectories of patients’ movements, with a starting and finishing point. Instead, they are endless loops of movements” [47].

Our results provide a particularly thorough description of how participants move from a state of total dependency during hospitalization regarding their medication management to a sudden and complete autonomy after hospital discharge impacting medication management, understanding, and adherence in the first days after discharge for some participants. Several qualitative studies have described the lack of shared decision-making and the loss of patient autonomy during hospitalization, which had an impact on self-management and created conflicts with healthcare professionals [75, 81, 84]. Our study also highlights nuanced patient experiences, including varying levels of patient needs, involvement, and proactivity during hospitalization and outpatient care, and our results contribute to capturing different perspectives that contrast with some literature that often portrays patients as more passive recipients of care [14, 15, 74, 75]. Shared decision-making and proactive medication are key elements as they contribute to a smoother transition and better outcomes for patients post-discharge [85–87].

Consistent with the literature, the study identifies some challenges in medication initiation post-discharge [16, 17, 88] but our results also describe how daily routine rapidly takes over, either solidifying adherence behavior or generating barriers to medication adherence. Participants’ nonadherence prior to hospitalization was a factor influencing participants’ adherence post-hospitalization and this association should be further investigated, as literature showed that hospitalized patients have high scores of non-adherence [89]. Mortel et al. showed that more than 20% of discharged patients stopped their medications earlier than agreed with the physician and 25% adapted their medication intake [90]. Furthermore, patients who self-managed their medications had a lower perception of the necessity of their medication than patients who received help, which could negatively impact medication adherence [91]. Although participants in our study had high BMQ scores for necessity and lower scores for concerns, some participants expressed doubts about the need for their medications and a lack of motivation a few weeks after discharge. Targeted pharmacy interventions for newly prescribed medications have been shown to improve medication adherence, and hospital discharge is an opportune moment to implement this service [92, 93].

Many medication changes were made during the transition of care (a median number of 7 changes during hospitalization and 7 changes during the two months after discharge), especially medication additions during hospitalization and interruptions after hospitalization. While medication changes during hospitalization are well described, the many changes following discharge are less discussed [7, 94]. A Danish study showed that approximately 65% of changes made during hospitalization were accepted by primary healthcare professionals but only 43% of new medications initiated during hospitalization were continued after discharge [95]. The numerous changes after discharge may be caused by unnecessary intensification of medications during hospitalization, delayed discharge letters, lack of standardized procedures, miscommunication, patient self-management difficulties, or in response to an acute situation [96–98]. During the transition of care, in our study, both new and experienced participants were faced with difficulties in managing and understanding medication changes, either for newly prescribed medication or changes in previous medications. Such difficulties corroborate the findings of the literature [9, 10, 47] and our results showed that the lack of understanding during hospitalization led to participants having questions about their medications, even weeks after discharge. Particular attention should be given to patients’ understanding of medication changes jointly by physicians, nurses and pharmacists during the transition of care and in the months that follow as medications are likely to undergo as many changes as during hospitalization.

### Implication for practice and future research

The patients’ perspectives in this study showed, at a system level, that there was a lack of standardization in healthcare professional practices regarding medication dispensing and follow-up. For now, in Switzerland, there are no official guidelines on medication prescription and dispensation during the transition of care although some international guidelines have been developed for outpatient healthcare professionals [3, 99–102]. Here are some suggestions for improvement arising from our results. Patients should be included as partners and healthcare professionals should systematically assess (i) previous medication adherence, (ii) patients’ desired level of involvement and (iii) their needs for information during hospitalization. Hospital discharge processes should be routinely implemented to standardize hospital discharge preparation, medication prescribing, and dispensing. Discharge from the hospital should be planned with community pharmacies to ensure that all medications are available and, if necessary, doses of medications should be supplied by the hospital to bridge the gap. A partnership with outpatient healthcare professionals, such as general practitioners, community pharmacists, and homecare nurses, should be set up for effective asynchronous interprofessional collaboration to consolidate patients’ medication management, knowledge, and adherence, as well as to monitor signs of deterioration or adverse drug events.

Future research should consolidate our first attempt to develop a framework to better characterize medication at the transition of care, using Fig. 2  as a starting point. Contextualized interventions, co-designed by health professionals, patients and stakeholders, should be tested in a hybrid implementation study to test the implementation and effectiveness of the intervention for the health system [103].

### Limitations

This study has some limitations. First, the transcripts were validated for accuracy by the interviewer but not by a third party, which could have increased the robustness of the transcription. Nevertheless, the interviewer followed all methodological recommendations for transcription. Second, patient inclusion took place during the COVID-19 pandemic, which may have had an impact on patient care and the availability of healthcare professionals. Third, we cannot guarantee the accuracy of some participants’ medication history before hospitalization, even though we contacted the participants’ main pharmacy, as participants could have gone to different pharmacies to obtain their medications. Fourth, our findings may not be generalizable to other populations and other healthcare systems because some issues may be specific to multimorbid patients with type 2 diabetes or to the Swiss healthcare setting. Nevertheless, issues encountered by our participants regarding their medications correlate with findings in the literature. Fifth, only 15 out of 21 participants took part in all the interviews, but most participants took part in at least three interviews and data saturation was reached. Lastly, by its qualitative and longitudinal design, it is possible that the discussion during interviews and participants’ reflections between interviews influenced participants’ management, knowledge, and adherence, even though this study was observational, and no advice or recommendations were given by the interviewer during interviews.

## Conclusions

Discharged patients are willing to take steps to better manage, understand, and adhere to their medications, yet they are also faced with difficulties in the hospital and outpatient care. Furthermore, extensive changes in medications not only occur during hospitalization but also during the two months following hospital discharge, for which healthcare professionals should give particular attention. The different degrees of patients’ involvement, needs and resources should be carefully considered to enable them to better manage, understand and adhere to their medications. At a system level, patients’ experiences revealed a lack of standardization of medication practices during the transition of care. The healthcare system should provide the ecosystem needed for healthcare professionals responsible for or involved in the management of patients’ medications during the hospital stay, discharge, and outpatient care to standardize their practices while considering the patient as an active partner.

### Electronic supplementary material

Below is the link to the electronic supplementary material.

Supplementary Material 1

Supplementary Material 2

Supplementary Material 3

## Data Availability

The anonymized quantitative survey datasets and the qualitative codes are available in French from the corresponding author on reasonable request.

## Abbreviations

ADE: adverse drug events
AVAS: Adherence Visual Analogue Scale
BMQ: Belief in Medication Questionnaire
COREQ: Consolidated Criteria for Reporting Qualitative Research
CRFs: case report form
SD: standard deviation
WHO: World Health Organization

## References

[1] American Academy of Family Physician. Continuity of Care, Definition of 2020. Accessed 10 July 2022 https://www.aafp.org/about/policies/all/continuity-of-care-definition.html

[2] Kripalani S, LeFevre F, Phillips CO, Williams MV, Basaviah P, Baker DW (2007). Deficits in communication and information transfer between hospital-based and primary care physicians: implications for patient safety and continuity of care. JAMA.

[3] World Health Organization (WHO). Medication Safety in Transitions of Care. 2019.

[4] Forster AJ, Murff HJ, Peterson JF, Gandhi TK, Bates DW (2003). The incidence and severity of adverse events affecting patients after discharge from the hospital. Ann Intern Med.

[5] Krumholz HM (2013). Post-hospital syndrome–an acquired, transient condition of generalized risk. N Engl J Med.

[6] Banholzer S, Dunkelmann L, Haschke M, Derungs A, Exadaktylos A, Krähenbühl S (2021). Retrospective analysis of adverse drug reactions leading to short-term emergency hospital readmission. Swiss Med Wkly.

[7] Blozik E, Signorell A, Reich O (2016). How does hospitalization affect continuity of drug therapy: an exploratory study. Ther Clin Risk Manag.

[8] Allen J, Hutchinson AM, Brown R, Livingston PM (2018). User experience and care for older people transitioning from hospital to home: patients’ and carers’ perspectives. Health Expect.

[9] Daliri S, Bekker CL, Buurman BM, Scholte Op Reimer WJM, van den Bemt BJF, Karapinar-Çarkit F (2019). Barriers and facilitators with medication use during the transition from hospital to home: a qualitative study among patients. BMC Health Serv Res.

[10] Bekker CL, Mohsenian Naghani S, Natsch S, Wartenberg NS, van den Bemt BJF (2020). Information needs and patient perceptions of the quality of medication information available in hospitals: a mixed method study. Int J Clin Pharm.

[11] Foulon V, Wuyts J, Desplenter F, Spinewine A, Lacour V, Paulus D (2019). Problems in continuity of medication management upon transition between primary and secondary care: patients’ and professionals’ experiences. Acta Clin Belgica: Int J Clin Lab Med.

[12] Micheli P, Kossovsky MP, Gerstel E, Louis-Simonet M, Sigaud P, Perneger TV (2007). Patients’ knowledge of drug treatments after hospitalisation: the key role of information. Swiss Med Wkly.

[13] Ziaeian B, Araujo KL, Van Ness PH, Horwitz LI (2012). Medication reconciliation accuracy and patient understanding of intended medication changes on hospital discharge. J Gen Intern Med.

[14] Allen J, Hutchinson AM, Brown R, Livingston PM (2016). User experience and care integration in Transitional Care for older people from hospital to home: a Meta-synthesis. Qual Health Res.

[15] Mackridge AJ, Rodgers R, Lee D, Morecroft CW, Krska J (2018). Cross-sectional survey of patients’ need for information and support with medicines after discharge from hospital. Int J Pharm Pract.

[16] Mulhem E, Lick D, Varughese J, Barton E, Ripley T, Haveman J (2013). Adherence to medications after hospital discharge in the elderly. Int J Family Med.

[17] Fallis BA, Dhalla IA, Klemensberg J, Bell CM (2013). Primary medication non-adherence after discharge from a general internal medicine service. PLoS ONE.

[18] Zhou L, Rupa AP (2018). Categorization and association analysis of risk factors for adverse drug events. Eur J Clin Pharmacol.

[19] Moreau-Gruet F. La multimorbidité chez les personnes de 50 ans et plus. Résultats basés sur l’enqête SHARE (Survey of Health, Ageing and Retirement in Europe. Obsan Bulletin 4/2013. 2013(Neuchâtel: OBservatoire suisse de la santé).

[20] Iglay K, Hannachi H, Joseph Howie P, Xu J, Li X, Engel SS (2016). Prevalence and co-prevalence of comorbidities among patients with type 2 diabetes mellitus. Curr Med Res Opin.

[21] Sibounheuang P, Olson PS, Kittiboonyakun P (2020). Patients’ and healthcare providers’ perspectives on diabetes management: a systematic review of qualitative studies. Res Social Adm Pharm.

[22] Müller-Wieland D, Merkel M, Hamann A, Siegel E, Ottillinger B, Woker R (2018). Survey to estimate the prevalence of type 2 diabetes mellitus in hospital patients in Germany by systematic HbA1c measurement upon admission. Int J Clin Pract.

[23] Blanc AL, Fumeaux T, Stirnemann J, Dupuis Lozeron E, Ourhamoune A, Desmeules J (2019). Development of a predictive score for potentially avoidable hospital readmissions for general internal medicine patients. PLoS ONE.

[24] Hansen LO, Greenwald JL, Budnitz T, Howell E, Halasyamani L, Maynard G (2013). Project BOOST: effectiveness of a multihospital effort to reduce rehospitalization. J Hosp Med.

[25] Khalid JM, Raluy-Callado M, Curtis BH, Boye KS, Maguire A, Reaney M (2014). Rates and risk of hospitalisation among patients with type 2 diabetes: retrospective cohort study using the UK General Practice Research Database linked to English Hospital Episode statistics. Int J Clin Pract.

[26] Lussier ME, Evans HJ, Wright EA, Gionfriddo MR (2020). The impact of community pharmacist involvement on transitions of care: a systematic review and meta-analysis. J Am Pharm Assoc.

[27] van der Heijden A, de Bruijne MC, Nijpels G, Hugtenburg JG (2019). Cost-effectiveness of a clinical medication review in vulnerable older patients at hospital discharge, a randomized controlled trial. Int J Clin Pharm.

[28] Bingham J, Campbell P, Schussel K, Taylor AM, Boesen K, Harrington A (2019). The Discharge Companion Program: an interprofessional collaboration in Transitional Care Model Delivery. Pharm (Basel).

[29] Farris KB, Carter BL, Xu Y, Dawson JD, Shelsky C, Weetman DB (2014). Effect of a care transition intervention by pharmacists: an RCT. BMC Health Serv Res.

[30] Meslot C, Gauchet A, Hagger MS, Chatzisarantis N, Lehmann A, Allenet B (2017). A Randomised Controlled Trial to test the effectiveness of planning strategies to improve Medication Adherence in patients with Cardiovascular Disease. Appl Psychol Health Well Being.

[31] Garnier A, Rouiller N, Gachoud D, Nachar C, Voirol P, Griesser AC (2018). Effectiveness of a transition plan at discharge of patients hospitalized with heart failure: a before-and-after study. ESC Heart Fail.

[32] Daliri S, Bekker CL, Buurman BM, Scholte Op Reimer WJM, van den Bemt BJF, Karapinar-Çarkit F. Medication management during transitions from hospital to home: a focus group study with hospital and primary healthcare providers in the Netherlands. Int J Clin Pharm. 2020. 10.1007/s11096-020-01189-9 33128661

[33] Hansen LO, Young RS, Hinami K, Leung A, Williams MV (2011). Interventions to reduce 30-day rehospitalization: a systematic review. Ann Intern Med.

[34] Leppin AL, Gionfriddo MR, Kessler M, Brito JP, Mair FS, Gallacher K (2014). Preventing 30-day hospital readmissions: a systematic review and meta-analysis of randomized trials. JAMA Intern Med.

[35] Donzé J, John G, Genné D, Mancinetti M, Gouveia A, Méan M et al. Effects of a Multimodal Transitional Care Intervention in patients at high risk of readmission: the TARGET-READ Randomized Clinical Trial. JAMA Intern Med. 2023. 10.1001/jamainternmed.2023.0791 PMC10152373 37126338

[36] Rodrigues CR, Harrington AR, Murdock N, Holmes JT, Borzadek EZ, Calabro K (2017). Effect of pharmacy-supported transition-of-care interventions on 30-Day readmissions: a systematic review and Meta-analysis. Ann Pharmacother.

[37] Lam MYY, Dodds LJ, Corlett SA (2019). Engaging patients to access the community pharmacy medicine review service after discharge from hospital: a cross-sectional study in England. Int J Clin Pharm.

[38] Hossain LN, Fernandez-Llimos F, Luckett T, Moullin JC, Durks D, Franco-Trigo L (2017). Qualitative meta-synthesis of barriers and facilitators that influence the implementation of community pharmacy services: perspectives of patients, nurses and general medical practitioners. BMJ Open.

[39] En-Nasery-de Heer S, Uitvlugt EB, Bet PM, van den Bemt BJF, Alai A, van den Bemt P et al. Implementation of a pharmacist-led transitional pharmaceutical care programme: process evaluation of medication actions to reduce hospital admissions through a collaboration between Community and Hospital pharmacists (MARCH). J Clin Pharm Ther. 2022. 10.1111/jcpt.13645 PMC9544789 35306683

[40] Morris ZS, Wooding S, Grant J (2011). The answer is 17 years, what is the question: understanding time lags in translational research. J R Soc Med.

[41] De Geest S, Zúñiga F, Brunkert T, Deschodt M, Zullig LL, Wyss K (2020). Powering Swiss health care for the future: implementation science to bridge the valley of death. Swiss Med Wkly.

[42] Noonan VK, Lyddiatt A, Ware P, Jaglal SB, Riopelle RJ, Bingham CO 3, et al. Montreal Accord on patient-reported outcomes (PROs) use series - paper 3: patient-reported outcomes can facilitate shared decision-making and guide self-management. J Clin Epidemiol. 2017;89:125–35. 10.1016/j.jclinepi.2017.04.017 28433671

[43] Hesselink G, Schoonhoven L, Barach P, Spijker A, Gademan P, Kalkman C (2012). Improving patient handovers from hospital to primary care: a systematic review. Ann Intern Med.

[44] (OFSP) Interprofessionnalité dans le domaine de la santé Soins ambulatoire. Accessed 4 January 2024. https://www.bag.admin.ch/bag/fr/home/strategie-und-politik/nationale-gesundheitspolitik/foerderprogramme-der-fachkraefteinitiative-plus/foerderprogramme-interprofessionalitaet.html

[45] Mitchell SE, Laurens V, Weigel GM, Hirschman KB, Scott AM, Nguyen HQ (2018). Care transitions from patient and caregiver perspectives. Ann Fam Med.

[46] Davoody N, Koch S, Krakau I, Hägglund M (2016). Post-discharge stroke patients’ information needs as input to proposing patient-centred eHealth services. BMC Med Inf Decis Mak.

[47] Ozavci G, Bucknall T, Woodward-Kron R, Hughes C, Jorm C, Joseph K (2021). A systematic review of older patients’ experiences and perceptions of communication about managing medication across transitions of care. Res Social Adm Pharm.

[48] Fylan B, Armitage G, Naylor D, Blenkinsopp A (2018). A qualitative study of patient involvement in medicines management after hospital discharge: an under-recognised source of systems resilience. BMJ Qual Saf.

[49] Fylan B, Marques I, Ismail H, Breen L, Gardner P, Armitage G (2019). Gaps, traps, bridges and props: a mixed-methods study of resilience in the medicines management system for patients with heart failure at hospital discharge. BMJ Open.

[50] Brandberg C, Ekstedt M, Flink M (2021). Self-management challenges following hospital discharge for patients with multimorbidity: a longitudinal qualitative study of a motivational interviewing intervention. BMJ Open.

[51] Lawton J, Rankin D, Peel E, Douglas M (2009). Patients’ perceptions and experiences of transitions in diabetes care: a longitudinal qualitative study. Health Expect.

[52] Mabire C, Bachnick S, Ausserhofer D, Simon M (2019). Patient readiness for hospital discharge and its relationship to discharge preparation and structural factors: a cross-sectional study. Int J Nurs Stud.

[53] Meyers DC, Durlak JA, Wandersman A (2012). The quality implementation framework: a synthesis of critical steps in the implementation process. Am J Community Psychol.

[54] Meyer-Massetti C, Hofstetter V, Hedinger-Grogg B, Meier CR, Guglielmo BJ (2018). Medication-related problems during transfer from hospital to home care: baseline data from Switzerland. Int J Clin Pharm.

[55] Neeman M, Dobrinas M, Maurer S, Tagan D, Sautebin A, Blanc AL (2017). Transition of care: a set of pharmaceutical interventions improves hospital discharge prescriptions from an internal medicine ward. Eur J Intern Med.

[56] Geese F, Schmitt KU. Interprofessional Collaboration in Complex Patient Care Transition: a qualitative multi-perspective analysis. Healthc (Basel). 2023;11(3). 10.3390/healthcare11030359 PMC9914692 36766934

[57] Craig P, Dieppe P, Macintyre S, Michie S, Nazareth I, Petticrew M (2013). Developing and evaluating complex interventions: the new Medical Research Council guidance. Int J Nurs Stud.

[58] Thomson R, Plumridge L, Holland J, Editorial (2003). Int J Soc Res Methodol.

[59] Audulv Å, Hall EOC, Kneck Å, Westergren T, Fegran L, Pedersen MK (2022). Qualitative longitudinal research in health research: a method study. BMC Med Res Methodol.

[60] Kim H, Sefcik JS, Bradway C (2017). Characteristics of qualitative descriptive studies: a systematic review. Res Nurs Health.

[61] Sandelowski M (2000). Whatever happened to qualitative description?. Res Nurs Health.

[62] Bradshaw C, Atkinson S, Doody O (2017). Employing a qualitative description Approach in Health Care Research. Glob Qual Nurs Res.

[63] Bellone JM, Barner JC, Lopez DA (2012). Postdischarge interventions by pharmacists and impact on hospital readmission rates. J Am Pharm Assoc (2003).

[64] Hennink MM, Kaiser BN, Marconi VC (2016). Code saturation versus meaning saturation: how many interviews are Enough?. Qual Health Res.

[65] World Health Organization. Adherence to long-term therapies: evidence for action. 2003.

[66] Fisher JD, Fisher WA, Amico KR, Harman JJ (2006). An information-motivation-behavioral skills model of adherence to antiretroviral therapy. Health Psychol.

[67] Bandura A (1998). Health promotion from the perspective of social cognitive theory. Psychol Health.

[68] ShiftEUROQOL Research FOndation EQ 5D Instruments. Accessed 30 July 2022 https://euroqol.org/eq-5d-instruments/sample-demo/

[69] Jeppesen KM, Coyle JD, Miser WF (2009). Screening questions to predict limited health literacy: a cross-sectional study of patients with diabetes mellitus. Ann Fam Med.

[70] Giordano TP, Guzman D, Clark R, Charlebois ED, Bangsberg DR (2004). Measuring adherence to antiretroviral therapy in a diverse population using a visual analogue scale. HIV Clin Trials.

[71] Horne R, Weinman J, Hankins M (1999). The beliefs about medicines questionnaire: the development and evaluation of a new method for assessing the cognitive representation of medication. Psychol Health.

[72] Horne R, Chapman SC, Parham R, Freemantle N, Forbes A, Cooper V (2013). Understanding patients’ adherence-related beliefs about medicines prescribed for long-term conditions: a meta-analytic review of the necessity-concerns Framework. PLoS ONE.

[73] Braun V, Clarke V (2006). Using thematic analysis in psychology. Qualitative Res Psychol.

[74] Waibel S, Henao D, Aller M-B, Vargas I, Vázquez M-L (2011). What do we know about patients’ perceptions of continuity of care? A meta-synthesis of qualitative studies. Int J Qual Health Care.

[75] Rognan SE, Jørgensen MJ, Mathiesen L, Druedahl LC, Lie HB, Bengtsson K (2023). The way you talk, do I have a choice?’ Patient narratives of medication decision-making during hospitalization. Int J Qualitative Stud Health Well-being.

[76] Michel B, Hemery M, Rybarczyk-Vigouret MC, Wehrle P, Beck M (2016). Drug-dispensing problems community pharmacists face when patients are discharged from hospitals: a study about 537 prescriptions in Alsace. Int J Qual Health Care.

[77] Bruhwiler LD, Hersberger KE, Lutters M (2017). Hospital discharge: what are the problems, information needs and objectives of community pharmacists? A mixed method approach. Pharm Pract (Granada).

[78] Knight DA, Thompson D, Mathie E, Dickinson A (2013). Seamless care? Just a list would have helped!’ Older people and their carer’s experiences of support with medication on discharge home from hospital. Health Expect.

[79] Gualandi R, Masella C, Viglione D, Tartaglini D (2019). Exploring the hospital patient journey: what does the patient experience?. PLoS ONE.

[80] Norberg H, Håkansson Lindqvist M, Gustafsson M (2023). Older individuals’ experiences of Medication Management and Care after Discharge from Hospital: an interview study. Patient Prefer Adherence.

[81] Jones KC, Austad K, Silver S, Cordova-Ramos EG, Fantasia KL, Perez DC (2023). Patient perspectives of the hospital discharge process: a qualitative study. J Patient Exp.

[82] Hesselink G, Flink M, Olsson M, Barach P, Dudzik-Urbaniak E, Orrego C (2012). Are patients discharged with care? A qualitative study of perceptions and experiences of patients, family members and care providers. BMJ Qual Saf.

[83] Murray SA, Kendall M, Carduff E, Worth A, Harris FM, Lloyd A (2009). Use of serial qualitative interviews to understand patients’ evolving experiences and needs. BMJ.

[84] Berger ZD, Boss EF, Beach MC (2017). Communication behaviors and patient autonomy in hospital care: a qualitative study. Patient Educ Couns.

[85] Davis RE, Jacklin R, Sevdalis N, Vincent CA (2007). Patient involvement in patient safety: what factors influence patient participation and engagement?. Health Expect.

[86] Greene J, Hibbard JH (2012). Why does patient activation matter? An examination of the relationships between patient activation and health-related outcomes. J Gen Intern Med.

[87] Mitchell SE, Gardiner PM, Sadikova E, Martin JM, Jack BW, Hibbard JH (2014). Patient activation and 30-day post-discharge hospital utilization. J Gen Intern Med.

[88] Weir DL, Motulsky A, Abrahamowicz M, Lee TC, Morgan S, Buckeridge DL (2020). Failure to follow medication changes made at hospital discharge is associated with adverse events in 30 days. Health Serv Res.

[89] Kripalani S, Goggins K, Nwosu S, Schildcrout J, Mixon AS, McNaughton C (2015). Medication nonadherence before hospitalization for Acute Cardiac events. J Health Commun.

[90] Mortelmans L, De Baetselier E, Goossens E, Dilles T. What happens after Hospital Discharge? Deficiencies in Medication Management encountered by geriatric patients with polypharmacy. Int J Environ Res Public Health. 2021;18(13). 10.3390/ijerph18137031 PMC8293803 34209384

[91] Mortelmans L, Goossens E, Dilles T (2022). Beliefs about medication after hospital discharge in geriatric patients with polypharmacy. Geriatr Nurs.

[92] Bandiera C, Ribaut J, Dima AL, Allemann SS, Molesworth K, Kalumiya K et al. Swiss Priority setting on implementing Medication Adherence interventions as Part of the European ENABLE COST action. Int J Public Health. 2022;67. 10.3389/ijph.2022.1605204 PMC9411421 36032275

[93] Elliott R, Boyd M, Nde S. at e. Supporting adherence for people starting a new medication for a long-term condition through community pharmacies: a pragmaticrandomised controlled trial of the New Medicine Service. 2015.

[94] Grimmsmann T, Schwabe U, Himmel W (2007). The influence of hospitalisation on drug prescription in primary care–a large-scale follow-up study. Eur J Clin Pharmacol.

[95] Larsen MD, Rosholm JU, Hallas J (2014). The influence of comprehensive geriatric assessment on drug therapy in elderly patients. Eur J Clin Pharmacol.

[96] Viktil KK, Blix HS, Eek AK, Davies MN, Moger TA, Reikvam A (2012). How are drug regimen changes during hospitalisation handled after discharge: a cohort study. BMJ Open.

[97] Strehlau AG, Larsen MD, Søndergaard J, Almarsdóttir AB, Rosholm J-U (2018). General practitioners’ continuation and acceptance of medication changes at sectorial transitions of geriatric patients - a qualitative interview study. BMC Fam Pract.

[98] Anderson TS, Lee S, Jing B, Fung K, Ngo S, Silvestrini M (2020). Prevalence of diabetes medication intensifications in older adults discharged from US Veterans Health Administration Hospitals. JAMA Netw Open.

[99] Royal Pharmaceutical Society. Keeping patients safewhen they transfer between care providers– getting the medicines right June 2012. Accessed 27 October 2023 https://www.rpharms.com/Portals/0/RPS%20document%20library/Open%20access/Publications/Keeping%20patients%20safe%20transfer%20of%20care%20report.pdf

[100] International Pharmaceutical Federation (FIP). Medicines reconciliation: A toolkit for pharmacists. Accessed 23 September 2023 https://www.fip.org/file/4949

[101] Californian Pharmacist Assiociation Transitions of Care Resource Guide. https://cdn.ymaws.com/www.cshp.org/resource/resmgr/Files/Practice-Policy/For_Pharmacists/transitions_of_care_final_10.pdf

[102] Royal Collegue of Physicians. Medication safety at hospital discharge: Improvement guide and resource. Accessed 18 September 2023 https://www.rcplondon.ac.uk/file/33421/download

[103] Douglas N, Campbell W, Hinckley J. Implementation science: buzzword or game changer. J Speech Lang Hear Res. 2015;58. 10.1044/2015_JSLHR-L-15-0302 26502033

