# Supplementary material for: Patient medication management, understanding and adherence during the transition from hospital to outpatient care - a qualitative longitudinal study in polymorbid patients with type 2 diabetes
Source: BMC Health Serv Res. 2024 May 13;24:620. doi: 10.1186/s12913-024-10784-9 (PMC11089680; doi:10.1186/s12913-024-10784-9)
Supplement: Supplementary file 1 — Supplementary Material 1 [file 12913_2024_10784_MOESM1_ESM.pdf]

Additional File 1 : Participants characteristics

	Inaugural diabetes	< 60 years	Men	Woman	>1 hospitalization 6 months	>10 med.
Inaugural diabetes						
< 60 years	P5, P6, P16					
Male	P5,P6,P7, P12,P16	P5,P6,P10, P11,P16				
Female	P1,P3,P14,P23	P4				
>1 hospitalization 6 months	P6,P16,P23	P6,P16	P6,P9,p16	P17,P23		
>10 médicamentions	P3,P23	P4,p5,p6	P13,p20	P2,p4, P17, p22,p23	P17,P23	
Reason for hospitalization: diabetes	P1,P3,P5,P6, P12,P14,P23	P1,P2,P3,P13 P23	P5,P6,12,13	P1,P2,P3, P14,P23	P6,P23	
