# Supplementary material for: Patient medication management, understanding and adherence during the transition from hospital to outpatient care - a qualitative longitudinal study in polymorbid patients with type 2 diabetes
Source: BMC Health Serv Res. 2024 May 13;24:620. doi: 10.1186/s12913-024-10784-9 (PMC11089680; doi:10.1186/s12913-024-10784-9)
Supplement: Supplementary file 2 — Supplementary Material 2 [file 12913_2024_10784_MOESM2_ESM.pdf]

Additional File 2- Interview guide translated from French to English

Interview guides were translated from French; these guides are not validated for English use.

Interview 1: D3

PATIENT N° : DATE :

PLACE: *home/telephone/video/CMU/other*

Category	Questions	comments
You've been out of the hospital for 3 days. If you agree, I'm going to ask you a few questions about your experience since leaving the hospital.		
Experience of returning home	How did your return home go?	
Preparing for discharge	Did your **medication change** between the time you came into the hospital and the time you left? **What did you understand** about these changes/new medications?	
	**How involved** were you in the **changes** and **decisions** made while you were in the hospital?	
	Looking back, how **would you describe** your **preparation** during your hospital stay in terms of managing your new treatment at home?	
	What would you have **known/Like to know** about your medicines **before going home**?	
I'd like to talk to you about your support at home		
Caregiver	Do you live **alone** or with/near relatives?	
	If alone: **how are you coping with** this situation? **What kind of help** would you need?	
	If close: to what extent do **your family and friends help you** return home and manage your medication?	
Discharge prescription	Did you (or a carer) **collect** the **medication** on **the discharge prescription**?	
	if not: **Why did** you not collect your medication?	

	if yes :<br>Can you **tell me more about** your time at the pharmacy?<br>**What did you find useful,** less useful, or even useless in what you told me?<br>What did **you miss** during your visit to the pharmacy?	
I'm now going to ask you a few questions about any health professionals you may have met since you got home.		
Health professionals	**Which healthcare professional(s)** have you met since leaving the hospital? *e.g. doctors (GP, specialist), pharmacists (local, on-call), nurses, physio, etc.*	
	**When** did you see it?	
	**Why did** you meet xxx?	
Change of medication	Could you show me your **medicines/a list of the medicines** you are taking at the moment?	
	Have any of your medications been **changed** or **stopped** since you returned home?	
	If so, which ones?<br>What did you **know about the changes**?<br>How do you feel about these changes (adaptation, change, satisfaction, confusion, etc.)?	
We're now going to take a look at how you manage your medicines on a day-to-day basis. Some patients report having difficulties with their medication, whether it's problems getting their medication, side effects, difficulties taking or using their medication, or other difficulties.		
Medication self-management	**What difficulties have** you encountered with your medicines/medical devices?<br>*Examples: logistics, time of administration, forgetfulness, AEs, interactions, difficulty of use, etc.*	
	If yes: In your opinion, what are the **causes of this/these difficulty**(s)?<br>**How** do these difficulties affect your medicine management?<br>Do you have any **solutions to** help you **overcome** these difficulties?	
	We've talked about the difficulties of managing certain medications. What helps you manage and take your medication?<br>*Examples: reminding you to use the telephone, putting medicines away in a particular place, rituals*	
We are coming to the end of our first interview:		
	Would you like to add anything?	

Notes

Interview 2: Day 10

PATIENT N°: DATE:
PLACE: *home/telephone/video/CMU/other*

**Category**	**Questions**	**Comment**
You've been back home for about ten days now. If you don't mind, I'd like to ask you a few questions.		
Introductory question	How have you been feeling since we last spoke?	
Health professionals	What healthcare **professional(s) have** you met since we last spoke? *e.g. doctors (GP, specialist), pharmacists (local, on-call), nurses, physio, etc.*	
	**When** did you see it?	
	**Why did** you meet xxx?	
We will now take a look at the medicines you are taking and how to manage them.		
Change of medication	Last time, we saw that you were taking :<br><br>What is the situation **today**?	
	Have there been any **changes** to your medication since we last spoke?	
	If so, which ones?<br>What did you **know about the changes**?<br>How do you feel about these changes (adaptation, change, satisfaction, confusion, etc.)?	
	You told me last time that you were taking :<br><br>and **you didn't mention it to me.** Why not?	
Information	With which professional(s) have you **discussed** your **medication**?	
	**What information did** you receive about your medication?	
	Would you like **more** information?	
	if yes :<br>Which ones?	

	**How can you** get them?<br>*Or* who or what could you turn to for this information?	
Medication advice	*We have talked about information that comes from health professionals (doctors, pharmacists).*<br>What about **advice** from **friends and family**?<br>What about the advice you read on the **internet**?<br>What kind of **emotions** did this trigger for you?	
Motivation	What do you **expect from** your medicines?	
	**What motivates** you to take your medication?	
	**How do those around** you **support** you in taking/managing your medication?	
In our last discussion, we talked about the difficulties and the factors that make it easier for you to take your medication. We're going to go over them again today.		
Medication self-management	*Presentation of a summary of the last discussion*. How are **things developing for** you at the moment?	
	If the situation is improving: what is **contributing to this improvement**? what have you done?<br>*If the difficulty is still present:* What are the **factors** or elements that make the **difficulty always** present? *or Why do* you think the situation is always difficult?	
	Since we last met, have there been any **new difficulties** with your medication?<br>*Example: logistics, time of intake, forgetfulness, AEs, interactions*	
	If yes: In your opinion, what are **the causes of** this/these difficulty(s)? How do these difficulties affect your medicine management?<br>Do you have any **solutions to** help you overcome these difficulties?	
	**What** helps you take/manage your medication?	
Adherence means taking your medicines as prescribed. There are many reasons why people do not necessarily take their medication every day, at the same time, and under the right conditions.		
Adherence	What is/are **the most difficult medication(s) for** you to take at the moment?	

	Can you tell me about a **time** when you **didn't take your** medication **or a time when it was difficult to** take your medication? How **often does** this type of situation arise? Under what **circumstances**?<br>Are there **other difficult situations**? If so, which ones?<br>Can you remember the **last time you didn**'t take your medicine(s)?<br>How did you **feel**?	
	What factors make it easier for you to take/manage your medicines (e.g. practical, emotional, linked to the treatment, to the healthcare system)?	
We are coming to the end of our interview:		
	What could be put in **place** to make your day-to-day medication **management easier**?	
	What do you think of the usefulness of **electronic tools**?<br>*e.g. application, alarm, website*	
	Would you like to add anything?	

Notes:

Interview 3: D30

PATIENT N°: DATE:

PLACE: *home/telephone/video/CMU/other*

Category	Questions	Comment
You've been back home for a month now. If you don't mind, I'd like to ask you a few questions.		
Introductory question	How have you been **feeling** since we last spoke?	
Health professionals	Which healthcare **professional(s)** have you met since we last spoke? *e.g. doctors (GP, specialist types), pharmacists (local, on-call), nurses, physio, etc.*	
	**When** did you see it?	
	**Why did** you meet xxx?	
We will now take a look at the medicines you are taking and how to manage them.		
Change of medication	Last time, we saw that you were **taking** :<br><br>What is the situation today?	
	**Have there been any changes in** your medication since we last spoke?	
	If so, which ones?<br>What did you **know about the changes**?<br>How do you feel about these changes (adaptation, change, satisfaction, confusion, etc.)?	
	If stopping medication: The last time you took :<br><br>and **you didn't mention it to me**. Why not?	
In our last discussion, we talked about the difficulties and factors that make it easier for you to take your medication. We're going to go over them again today.		
Medication self-management	*Presentation of a summary of the last discussion*. How are things **developing for** you at the moment?	
	If the situation is improving: what is **contributing to** this **improvement**? what have you done?<br><br>*If the difficulty is still present:* What are the **factors** or elements that make the **difficulty always present**? *or Why do* you think the situation is always difficult?	

	Since we last met, have there been any **new difficulties** with your medication?	
	If yes :<br>In your opinion, what are **the causes of** this/these difficulty(s)?<br>**To what extent do** these difficulties affect your medicine management?<br>Do you have any solutions to help you overcome these difficulties?	
Storage of medicines	Where and how do you store your medicines?	
	Does this **help** you or make it **more difficult** to manage your medication? **Why or why not**?	
I'd now like to talk to you about the relationship you have with your doctor and your pharmacist, and how important that relationship is in managing your medicines.		
Healthcare professionals	Since your return home, could you describe a **situation/interaction** with a healthcare professional that you think **went well** and why it was beneficial to you?	
	What about a situation that **didn't go so well**?	
	How important is your **relationship** with your **GP in terms of** your day-to-day use of medication?	
Pharmacy	Do you always go to the **same** pharmacy?	
	If so, why **are you loyal to** this pharmacy?<br>*If not,* why are **you changing pharmacies**?	
	What **role** does **the pharmacist play** in **managing your medicines**?	
	**How** can the **pharmacist** better help you **manage/understand** your medicines?	
	How would you describe the **communication** and information sharing between your **pharmacist and your doctor**?	
We are coming to the end of our interview:		
	Would you like to add anything?	

Notes

Interview 4: Day 60

PATIENT N°: DATE: PLACE: *home/telephone/video/CMU/other*

Categories	Questions	Comment
You've been back home for almost 2 months now. This is our last interview together. If you don't mind, I'd like to ask you a few questions.		
Introductory question	**How have** you been **feeling** since we last spoke?	
Health professionals	Which **healthcare professional(s)** have you met since we last spoke?	
	**When** did you meet him?	
	**Why did** you meet xxx?	
We will now look together at the medicines you are taking and how they are managed.		
Change of medication	Last time, we saw that you were **taking** :<br><br>What is the situation **today**?	
	**Have there been any changes in** your medication since we last spoke?	
	If so, which ones?<br>What did you **know about the changes**?<br>How do you feel about these changes (adaptation, change, satisfaction, confusion, etc.)?	
	If stopping medication: The last time you **took** :<br><br>and you didn'**t mention it to** me. Why not?	
Information	With which professional(s) have you **discussed your medication**?	
	What **information did** you receive about your **medication**?	
	Would you like **more information**?	
	if yes :<br>**Which ones**?<br>**How** can you get them?<br>Who or what could you turn to for this information?	
Motivation	A few weeks ago, we talked about **your expectations of your medication**. I'd like to know **how your expectations of** your medication **have evolved**.	
	What about **your motivation to** take your medication?	

Medication self-management	*Presentation of a summary of the last discussion*. How are **things developing for** you at the moment?	
	If the situation is improving: what is **contributing to** this improvement? what have you done? *If the difficulty is still present:* What are the factors or elements that make **the difficulty always** present? *or Why do* you think the situation is always difficult?	
	Since we last met, have there been any **new difficulties** with your medication?	
	In your opinion, what are the **causes of this/these difficulty**(s)?	
	To what extent do these difficulties affect your medicine management?	
A few weeks ago we talked about therapeutic adherence, i.e. how you take your medication as prescribed. (Read a summary of the last interview)		
Therapeutic adherence	How are things **developing** for you? *If the situation is improving:* what is **contributing to** this improvement? *If adherence problems:* Which **medication**(s) is/are the **most difficult to take**? Can you tell me about a time when it was **difficult to** take your medication(s)? How did it make you feel?	
Looking back on your journey from hospital discharge to now, 2 months later, I'd like to ask you a few questions about how you navigated the healthcare system and how your needs have changed.		
General view: route	What are **the greatest difficulties you have** encountered in **your journey** through the healthcare system **since leaving the** hospital? **With your medications?**	
	What **ideas would you have for** improving the route?	
General view: support	*We've talked a lot about the difficulties you've encountered and your needs in terms of information, skills, and motivation about your medication.* How **would you describe the evolution of your needs** in terms of **self-management of your medication** from the time you returned home until now?	
	**How** have healthcare **professionals supported** you to **meet your** treatment **needs** over the last two months?	
	What could **improve this support** and collaboration?	
We are coming to the end of our interview:		
	Would you like to add anything?	

Notes
