# Supplementary material for: Patient medication management, understanding and adherence during the transition from hospital to outpatient care - a qualitative longitudinal study in polymorbid patients with type 2 diabetes
Source: BMC Health Serv Res. 2024 May 13;24:620. doi: 10.1186/s12913-024-10784-9 (PMC11089680; doi:10.1186/s12913-024-10784-9)
Supplement: Supplementary file 3 — Supplementary Material 3 [file 12913_2024_10784_MOESM3_ESM.pdf]

Additional File 3 – Self-reported adherence questionnaire and results

SELF REPORTED QUESTIONNAIRE

Please indicate below the three medications that cause you the most problems or questions and rate your adherence over the past week on a scale from 0 to 100% for each of these three medications.

Medication 1:

Medication 2:

Medication 3:

Concerning Medication 1: On a scale from 0 to 100 how would you rate taking this medication at the time and dose prescribed by your doctor over the last 7 days?

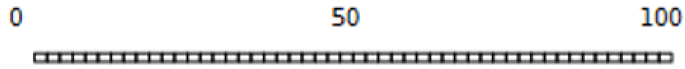

0 means that you have never taken this medication in the last 7 days. 100 means that you have taken this medication at the right time, frequency, and at the prescribed dose during the last 7 days.

DATA : PATIENT SELF-REPORTED ADHERENCE

Time points	Patient self-reported adherence [a], median (IQR)	Participants with 100% adherence, n (%)
At interview 2, n= 17	96 (89-100)	10 (58%)
At interview 3, n= 15	95 (83-100)	9 (60%)
At interview 4; n= 15	95 (85-100)	9 (60%)

[a]over the past week for their three most challenging medications; scale from 0 -100%
[b]for all three most challenging medications

The five most challenging medication classes according to participants were oral antidiabetics, antihypertensives, oral antiaggregants, subcutaneous antidiabetics and statins. A total of 61 % of the most challenging medications were newly introduced during the hospitalization.
